# A Case of Myxedema Coma Crisis Induced by Inhalation Injury

**DOI:** 10.7759/cureus.17049

**Published:** 2021-08-10

**Authors:** Muzamil Jawed, Julieta Osella, Donya Bani Hani

**Affiliations:** 1 Internal Medicine, Lincoln Medical Center, New York, USA

**Keywords:** myxedema coma, hypothyroid, inhalation, coma, smoke injury

## Abstract

Myxedema coma is a life-threatening, critical condition in which many organ systems can be severely affected. It is considered the most severe presentation of hypothyroidism and should be treated immediately. Here, we discuss the case of a 58-year-old patient who presented with altered mental status, bradycardia, and hypothermia, the critical characteristics considered in this disorder after inhalation injury. In order to avoid a fatal outcome, aggressive therapy should be initiated upon presentation. This case will depict the typical presentation, the specific cause pertinent to this patient’s condition, and the management of the acute condition of myxedema coma.

## Introduction

Myxedema coma (MC) is a rare and life-threatening illness, which should be recognized early to prevent morbidity and mortality. Patients with a history of endocrine disorders, especially those who are non-compliant with medications, or those who present after other organic causes are at high risk [1]. Here, we present the case of a 58-year-old male who was previously stable and later developed altered mental status, bradycardia, hypotension, and hypothermia after exposure to smoke and burns secondary to a house fire. Based on clinical presentation and elevated thyroid-stimulating hormone (TSH), a diagnosis of MC was made. Per literature review, inhalation-injury-induced MC has been rarely reported. This patient’s condition was initially refractory to supportive efforts until thyroid hormone was replaced aggressively. This case illustrates a rare etiology for this specific presentation and the immediate need for consideration of metabolic/endocrine causes when handling an unstable patient.

## Case presentation

**Figure 1:**
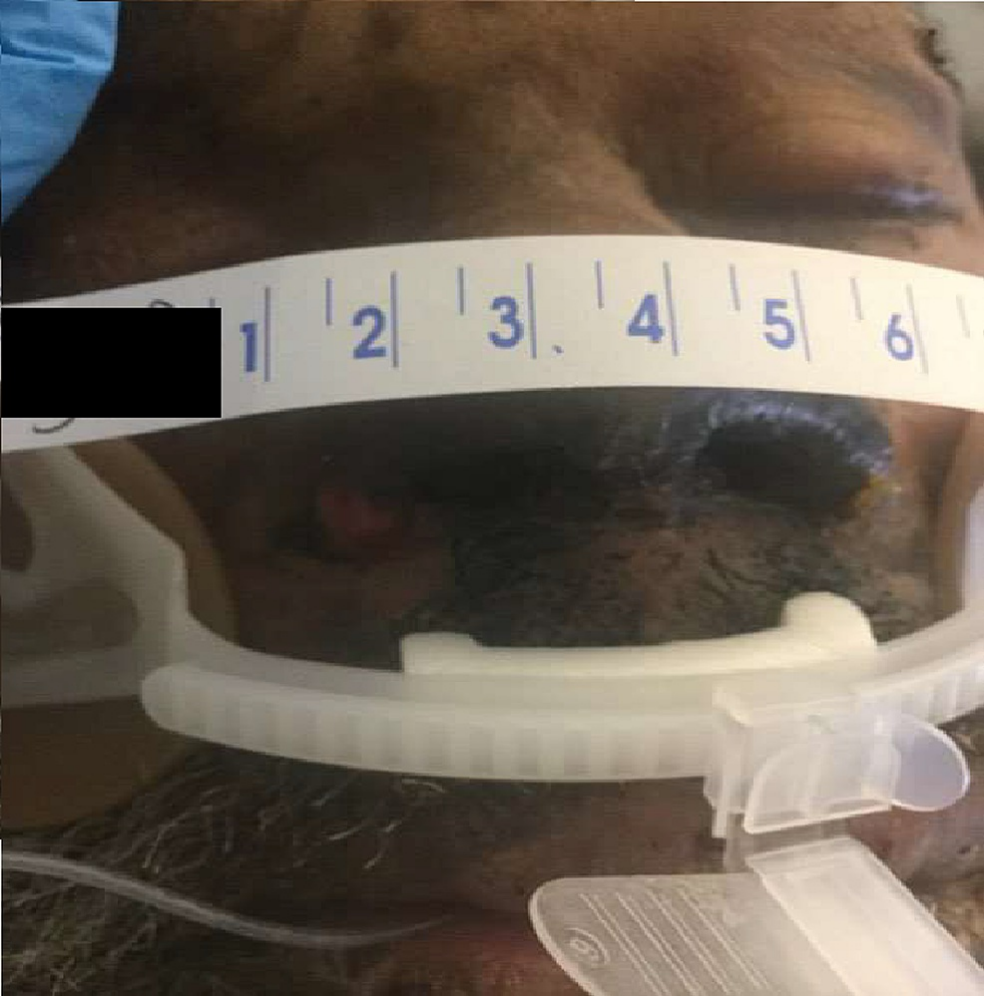
Blackened Nares Intubated patient noted with blackened nares. Measuring tape for scale.

A 58-year-old male with a past medical history of human immunodeficiency virus on highly active antiretroviral therapy, CD4 count 125, hepatitis C, chronic obstructive pulmonary disease on home oxygen, chronic kidney disease stage III, and hypothyroidism presented to the emergency department for shortness of breath after being rescued from a house fire. On arrival, the patient was noted to be uncomfortable and noted to have soot (Figure 1), vital signs noted as temperature of 98.5 F, heart rate of 89 beats per minute (BPM), respiratory rate 21, blood pressure 120/99 mmHg with oxygen saturation of 73% on room air. The patient was placed on non-rebreather mask; however, he did not tolerate and was consequently intubated for airway protection and acute hypoxemic respiratory failure with post-intubation arterial blood gas showing pH 7.34, pCO_2_ 71.3, and pO_2_ 391. The patient’s initial labs and analytics showed laboratory findings significant for macrocytic anemia, acute kidney injury on CKD with mild troponin leak and elevated pro brain natriuretic peptide, elevated carboxyhemoglobin levels (9.2%), and normal cyanide levels. Electrocardiography showed a normal sinus rhythm with chest X-ray showing right lower lobe infiltrate.

Hours after the intubation, the patient was found to be hypothermic to 95.0 F, bradycardic as low as 38 BPM, and hypotensive with a mean arterial pressure of <65 mmHg consequently requiring pressor support despite fluid resuscitation. Electrocardiogram showed sinus bradycardia with sinus arrhythmia, low-voltage QRS, and prolonged QT. The patient was actively rewarmed using warming blankets and sedation was discontinued to rule out any drug-induced cause of bradycardia but the patient remained bradycardic with poor mental status. The patient was spontaneously breathing, opened eyes, and was responsive to physical and verbal stimuli; however, he was not following any commands. Blood cultures and tracheal aspirate cultures were completed, and all were found to be negative. Thyroid function tests were done and the patient was found to have elevated TSH of 149 μIU/mL, depressed free triiodothyronine (T3) <0.39 ng/dL and depressed free thyroxine (T4) <0.1 ng/dL, and thyroid peroxidase antibody 52.3. Of note, there is no known TSH prior to this episode. Given the patient’s bradycardia, hypothermia, hypotension in the presence of a precipitating event (inhalational injury), hypoxemia, and hypercarbia, signs and symptoms were highly suggestive of MC.

The patient was started on hydrocortisone 100 mg IV three times daily with initial IV levothyroxine 200 mcg followed by 100 mcg along with T3 supplements 25 mcg via nasogastric tube for one dose followed by 12.5 mcg every 12 hours for a total of five days. Patient’s hypothermia and bradycardia improved. Regarding lab values, TSH improved from 149 to 51 and later 32 after three days of treatment. T3 and T4 improved to 1.92 and 0.5 ng/dL, respectively.

During the course of stay in the hospital, the patient clinically improved, was tapered off pressor support, and extubated successfully on day 8 of admission. Once tolerating oral medications, the patient was switched to levothyroxine 100 mcg daily and tapered off steroids. Based on the case presentation above, the patient was diagnosed with MC triggered by the house fire. Adequate treatment was provided to the patient and he improved significantly and favorably and was discharged home to follow up at the clinic. 

## Discussion

The exact etiology of altered mental status is often difficult to decipher. MC is a life-threatening illness presenting with altered mental status along with hypothermia and bradycardia. In our case, we present the unusual case precipitated by inhalation injury. Prompt recognition of MC is essential as rapid recognition and subsequent management prevents mortality. Despite this, diagnosis is often delayed due to rarity of the condition and the overlap of its manifestations with many other critical life-threatening conditions seen in the intensive care unit (ICU), such as shock, stroke, intoxication, decompensated heart failure, and hypothermia [2].

When a comatose patient, especially one with a known history of hypothyroidism, presents with one of the symptoms of the triad of hypothermia, hyponatremia, and hypercapnia, in which our patient exhibited all three, the suspicion for MC should be raised with immediate further work-up [1]. Work-up includes measurement of TSH, T4, T3, and cortisol levels [3]. There is no current validated scoring system for the diagnosis of MC, likely due to the rarity of the condition; however, one study has proposed a criterion based on the data from the retrospective analysis of 21 patients with a score of 60 or more highly suggestive of MC (Table 1) [4]. Based on this specific scoring system, our patient would receive a score of 120 placing him above the cutoff for suspecting MC [4]. This scoring system, however, has many limitations, including the small sample size of 21 patients, and sub-optimal specificity of 85%, restricting its use in everyday settings. The incidence of burn- or inhalation-injury-induced MC is quite rare, and upon further review, only one such case has been presented [5]. 

**Table 1:** Diagnostic Scoring System for Myxedema Coma [4] Abbreviations:  EKG = electrocardiogram; GFR= glomerular filtration rate. A score of 60 or higher is highly suggestive/diagnostic of myxedema coma. A score of 25-59 is suggestive of risk for myxedema coma, and a score below 25 is unlikely to indicate myxedema coma. Other EKG changes: QT prolongation, low-voltage complexes, bundle branch blocks, nonspecific ST-T changes, or heart blocks.

Diagnostic Scoring System for Myxedema Coma						
Component	Variable		Points	Component	Variable	Points
Thermoregulatory dysfunction	>35		0	Gastrointestinal	Anorexia/abdominal pain/constipation	5
	32-35		10		Decreased intestinal motility	15
	<32		20		Paralytic ileus	20
Central nervous system effects	Somnolent/lethargic		10	Precipitating event	Absent	0
	Obtunded		15		Present	10
	Stupor		20	Metabolic disturbances	Hyponatremia	10
	Coma/seizures		30		Hypoglycemia	10
Cardiovascular		Absent	0		Hypoxemia	10
	Bradycardia	50-59	10		Hypercarbia	10
		40-49	20		Decrease in GFR	10
	EKG changes		10	Others	Pleural effusions	10
	Pericardial effusion		10		Pulmonary edema	15
	Cardiomegaly		15		Hypotension	20

The treatment of MC involves a multidisciplinary approach including ICU admission with ventilatory and cardiovascular support. The mainstay of management involves hormone replacement with intravenous L-thyroxine. Although some controversy exists regarding the doses of levothyroxine (T4) and liothyronine (T3) due to the limited presentation of the illness, it is recommended that 100-150 μg levothyroxine is to be given initially followed by 75-100 μg daily along with steroids as another aspect of treatment that is often used due to the possibility of secondary hypothyroidism [6]. In critical illness, however, it has been noted that the rate of conversion between T4 to T3 is reduced, hence T3 supplement may be needed and should be given along with T4 during the first initial days of critical illness [7,8]. Our patient received a combination of treatment with both T4 and T3 supplements as well as steroids and improved favorably. This varies from a similar case with burn-injury-induced myxedema, which improved without T3 supplement although this case was non-intubated and had no other comorbidities [5]. Although, a factor to consider in this case would be the patient’s medication adherence. As the patient presented with altered mental status, history was unreliable when asked about thyroxine medication and compliance. 

## Conclusions

This case presents a rare life-threatening disorder triggered by inhalation of fumes. MC is a medical emergency and should be treated at a multidisciplinary level. It is vital that MC is considered in patients with a history of hypothyroidism with signs and symptoms of hypotension, hypothermia, bradycardia, or altered mental status. Early recognition and optimal treatment are key in order to prevent increasing morbidity and mortality.

## References

[1] Kwaku MP, Burman KD (2007). Myxedema coma. J Intensive Care Med.

[2] Fjølner J, Søndergaard E, Kampmann U, Nielsen S (2015). Complete recovery after severe myxoedema coma complicated by status epilepticus. BMJ Case Rep.

[3] Lklouk MA (2013). Myxoedema coma: a very rare presentation of severe hypothyroidism. J Med Cases.

[4] Popoveniuc G, Chandra T, Sud A (2014). A diagnostic scoring system for myxedema coma. Endocr Pract.

[5] Batista AS, Zane LL, Smith LM (2017). Burn-induced myxedema crisis. Clin Pract Cases Emerg Med.

[6] Wall CR (2000). Myxedema coma: diagnosis and treatment. Am Fam Physician.

[7] Wartofsky L, Burman KD (1982). Alterations in thyroid function in patients with systemic illness: the ‘euthyroid sick syndrome’. Endocr Rev.

[8] Ueda K, Kiyota A, Tsuchida M, Okazaki M, Ozaki N (2019). Successful treatment of myxedema coma with a combination of levothyroxine and liothyronine. Endocr J.

